# Sclerosing angiomatoid nodular transformation (SANT) of spleen mimicking a splenic abscess: Case report and review of the literature

**DOI:** 10.1016/j.ijscr.2019.02.015

**Published:** 2019-02-15

**Authors:** Massimo Capaldi, Pietro Fransvea, Gabriele Ricci, Francesca Stella, Silvia Trombetta, Saverio Cerasari, Carlo Cataldi, Sabrina Casale, Pierluigi Marini

**Affiliations:** a General and Emergency Surgery, St. Camillo Forlanini’s Hospital, Rome, Italy; b Faculty of Medicine and Psychology, “Sapienza” University of Rome, St. Andrea’s Hospital, Italy; c Pathology Unit, St. Camillo Forlanini’s Hospital, Rome, Italy

**Keywords:** SANT, Spleen, Abscess

## Abstract

**Introduction:**

SANT of the spleen is a benign lesion that does not recur after splenectomy with unknown etiology.

**Presentation of the case:**

We report a case of sclerosing angiomatoid nodular transformation (SANT) of the spleen mimicking a splenic abscess in a patient with imaging studies that failed to differentiate this lesion from other splenic lesions.

**Discussion:**

SANT of the spleen is a rare condition, with only less than 100 cases reported in the literature. Clinically, SANT is only a kind of described pathological diagnostic conception. As in our case, these splenic lesions are often incidental findings on imaging studies performed for other reasons.

**Conclusion:**

The patient was treated with splenectomy, which has proved to be both diagnostic and therapeutic. Although histology can lead to the diagnosis of vascular tumor, immunohistochemistry is the only way to confirm the diagnosis.

## Highlights

• Sclerosing angiomatoid nodular transformation (SANT) is a recently recognized benign, proliferative vascular lesion affecting the spleen.
• A limited number of cases are described in the worldwide literature.
• Further case studies are needed to elucidate the mechanism of SANT and its possible various etiologies.
• In the light of above splenectomy at now is the choice treatment both for diagnosis and treatment.
• This research did not receive any specific grant from funding agencies in the public, commercial, or not-for-profit sectors.

## 1 Introduction

Sclerosing angiomatoid nodular transformation (SANT) is a recently recognized benign, proliferative vascular lesion affecting the spleen with a limited number of cases described in the worldwide literature. In this report, we present a new case of SANT mimicking a splenic abscess that had been treated with splenectomy successfully. We hope this report will help to accumulate more experience for an accurate diagnosis and proper therapy of SANT. The work has been reported in line with the SCARE criteria [1].

## 2 Case report

**Fig. 1:**
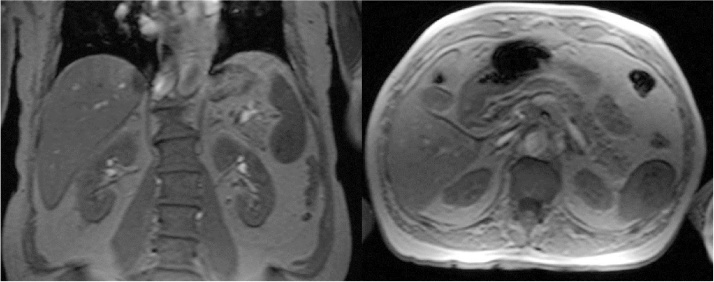
A mass of lower third of the spleen (55 mm of diameter) with a not uniform enhancement.

**Fig. 2:**
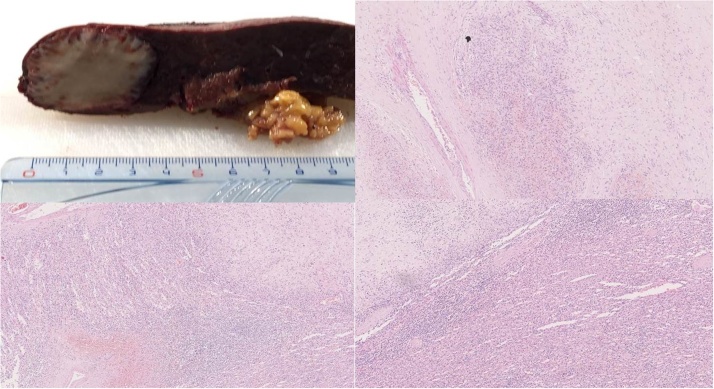
Splenice mass, “like capillary” vascular spaces surrounded by thick connective tissue.

A 67 Y old, caucasian male, patient was admitted to outpatients’ service of our Istitution showing an US and a CT scan. These diagnostic tests described a solid mass with a diameter of 55 mm localized at lower pole of the spleen. The imaging performed did not provide an unambiguous definition about the mass, so a MRI of the abdomen was also performed. Unfortunately, also MRI scan did not reveal any remarkable features, showing a mass of lower third of the spleen (55 mm of diameter) with a not uniform enhancement (Fig. 1). At the admission to ward, he denied any recent fever, allergy, chills, or changes in bowel habits. He had history of ischaemic cardiopathy with acute myocardial infarction five years before. Physical examination revealed no pathological findings. Laboratory values upon admission showed 15 g/dL haemoglobin, 45% hematocrit, 88 fL mean corpuscular volume (normal = 83–97), 31 pg mean corpuscular haemoglobin (normal = 27–33), 36 g/dl mean corpuscular haemoglobin concentration (normal = 32–36), 11,000 × 10*3/uL white blood cells (WBC) and C reactive protein (CRP) value was 5 mg/dl (normal value <0.5). The remaining laboratory data including electrolytes, liver function tests, urine analysis and coagulation factors were unremarkable. According to these findings, with the suspicious of splenic abscess, patients underwent surgical intervention with diagnostic and therapeutic intent. At surgery a little enlarged spleen with a mass located in lower pole was confirmed. The dimensions of the mass were approximately like a chicken egg with an hard, woody consistency. Upon this findings a splenectomy was performed. The post-operative course was complicated by mild fever (37.5–37.8 °C) between 3rd and 5th postoperative days. Due to this a CT scan was performed showing a fluid collection (diameter 10 cm) in splenic seat therefore at the same time a percutaneous drainage was placed and a full recovery was obtained in two days. The following postoperative course was uneventful and patients was discharged in postoperative day 7^th^. The pathological examination documented a splenic mass (4.5 × 3.5 cm) formed by many little nodules composed by “like capillary” vascular spaces surrounded by thick connective tissue. Immunohistochemical profiles was then performed and diagnosis of SANT was made (Fig. 2). The patient is asymptomatic and disease free at 3 years after surgery.

## 3 Discussion

Splenic lesions are commonly encountered and are often incidental in nature. Benign splenic vascular neoplasms include hemangioma, hamartoma, lymphangioma, extramedullary hematopoiesis (EMH), and sclerosing angiomatoid nodular transformation (SANT) [1]. SANT of the spleen is a rare condition, with only less than 100 cases reported in the literature. It was first described by Martel et al. in 2004, but recently more case reports are being published [2]. Martel et al. suggested that the nodules in SANT are derived from splenic red pulp and that they arise due to nodular transformation. These lesions were solitary, well circumscribed with a multinodular appearance, but without a capsule. Histological analysis revealed that the angiomatoid nodules were made up of loose connective tissue comprising a network (of varying densities) of aSMA + fusiform cells (probably myofibroblasts) and a rich network of capillaries [3]. Diebold et al. points out that SANT of the red pulp is a distinct benign pseudotumorous lesion of the spleen characterized by the presence of angiomatoid nodules [4]. Krishnan et al. as well as Martel et al. conducted immunohistochemistry for endothelial cell markers to distinguish neocapillaries that have endothelial cells with the same phenotype as normal cord capillaries and terminal arteries (CD34+, CD31+, CD8) from remnants of splenic sinusoids, which have endothelial cells producing CD31 and CD8, but not CD34 [5,6,2]. The classic appearance of SANT with regard to the immunohistochemical profiles includes three distinct types of blood vessels and endothelial cells stained with CD34, CD8 or CD31, respectively. The first type of vessels consists of well-formed cord capillaries in an organized lobular arrangement in endothelial cells positive for CD34 and CD31, but negative for CD8. The second type of vessels is consistent with splenic sinusoids and includes cells negative for CD34, but positive for CD31 and CD8. The third type consists of small veins that are arranged in intricate mesh-like patterns and include cells negative for CD34 and CD8, but positive for CD31. Focal expression of CD68 within the nodules may be seen, indicating an active phagocytic process in response to increased splenic activity; this further reinforces the non-neoplastic nature of SANT [[7], [8], [9]]. At now the mechanism underlying the formation of the angiomatoid nodules remains unknown. Some Authors hypothesize that tissue made up of myofibroblasts and neocapillaries develops in various pathological conditions [10]. Disturbed intrasplenic blood circulation may cause passive congestion of the red pulp. Such blood and lymph stasis may cause local metabolic changes (e.g. anoxia, decrease of pH), leading to damage of sinus endothelial cells. This may result in fibrin deposition and inflammation with production of connective tissue similar to granulation tissue occurring in repair processes [11,12]. Diebold et al. postulate that passive congestion of the red pulp may cause metabolic changes in those areas, damaging the sinus endothelial cells [4]. This may cause fibrin deposition and inflammation, as seen in granulation tissue. Martel et al. hypothesized that SANT was a response to stromal proliferation and that the internodular zones were very similar to inflammatory pseudotumor [2]. Given the similar immunohistochemical staining to that of splenic hamartoma, SANT may be on the spectrum of hamartomas because of the red pulp tissue composition, as theorized by Awamleh [13]. Kuo et al. have connected the plasma cells and stromal sclerosis present in SANT to IgG4-related sclerosing disease [14]. This idea is supported further by a recent report of three cases by Koreishi et al., in which all three cases had positive IgG4 plasma cells [15]. Clinically, SANT is only a kind of described pathological diagnostic conception. As in our case, these splenic lesions are often incidental findings on imaging studies performed for other reasons [16]. SANT is classically considered to be a female-predominant disease, with most of the patients in the 30- to 60-year age group. More recent finding [17,18] suggested that the gender predilection may be prone to be neutralized as more cases were described In the original report of Martel describing the largest series so far, one out of 25 patients presented with leukocytosis, polyclonal gammopathy, and raised erythrocyte sedimentation rate (ESR), one with fever, one with mild anaemia, and another one with pancytopenia with elevated ESR [2]. Most patients with SANT were asymptomatic at presentation, although some had non-specific abdominal pain and discomfort or splenomegaly. More clinical data are given in the Diebold study [4]. Three out of 16 patients in his study had some inflammatory condition and other 3 had anaemia. To the best of our knowledge, a clear association with other symptoms or complaints has not been described so far. The unique feature of our case were un increased with blood cell count and CRP value.

Because the differential diagnosis for a hypodense mass in the spleen includes lymphoma, metastasis, and benign lesions such as hamartoma, diagnostic studies are essential in the management. On computer tomography scans (CT) and MRI, SANT is a hypodense mass [[19], [20], [21]]. Karaosmanoglu et al. described MRI appearances on T1-weighted images as “spokes wheel pattern” [21,22]. Gutzeit et al. propose the use of contrast-enhanced ultrasonography (CEUS) to diagnose SANT, but the role of CEUS needs to be further evaluated as data is limited. There have been two reports of F-18 fluorodeoxyglucose (FDG)-avid splenic lesions found to be SANT lesions, however other authors have reported SANT cases without PET activity. Many patients presenting with solid lesions in the spleen will eventually be diagnosed with a malignant tumor, but it is difficult to rule out the possibility of a malignant neoplasm preoperatively based on conventional imaging studies. So it is mandatory to make pathologic confirmation for diagnosis and treatment of solid tumor. Weinreb et al. argue that due to its distinctive nodular pattern, lack of atypia, and unique immunohistochemical profile, that core biopsy can be used to distinguish SANT from other lesions in the differential diagnosis [23]. However, these procedure carries risks of bleeding and needle tract seeding [20]. Therefore, splenectomy may be the preferred modality to rule out malignancy or other pathological processes [24]. In addition the review of Cao et al. showed that patients with SANT, indeed, have a relatively high prevalence of coexistence with diseases at other organs [17,18]. Such a situation reminds clinicians and radiologists that, once a splenic lesion is discovered and, particularly, coexists synchronously with malignant tumors at other sizes (for which metastatic tumors cannot be ruled out, the possibility of patient with SANT should be considered integrating with imaging findings. Since the introduction of laparoscopic splenectomy by Delaitre and Maignien in 1992, now laparoscopic splenectomy has been recognized as a safe and effective treatment for hematologic disorders and other splenic diseases. Compared with open splenectomy, it is a safer procedure with the advantages of less postoperative pain and complications, early recovery from the procedure, shorter hospital stay, and a much smaller wound incision. Review of the existing literature revealed that only 8 cases of solitary SANT were treated with laparoscopic splenectomy. The treatment effect was similar to that by open operation according to the experience of follow up. In the cases reported to date, recurrence of SANT does not occur. More research about SANT is necessary, but as more cases are described, an etiology will likely be discovered.

## 4 Conclusion

The present study reports a novel case of SANT that underwent splenectomy successfully. Further case studies are needed to elucidate the mechanism of SANT and its possible various etiologies. In the light of above splenectomy at now is the choice treatment both for diagnosis and treatment.

## Conflicts of interest

The authors declare no potential financial conflict of interest related to this manuscript.

## Sources of funding

This research did not receive any specific grant from funding agencies in the public, commercial, or not-for-profit sectors.

## Ethical approval

The study is exempt from ethnical approval.

## Consent

Written consent was obtained by the patient.

## Author’s contribution

Study concept and design: P. Fransvea, M. Capaldi.

Data Collection: M. Capaldi.

Data Analysis and interpretation: P. Frasvea, G. Ricci.

Writing the paper: P. Fransvea, M. Capaldi.

Revision: P. Marini G. Rcci.

## Registration of research studies

Not needed.

## Guarantor

Massimo Capaldi MD is the Guarantor of the study.

## Provenance and peer review

Not commissioned, externally peer-reviewed.
